# Glucose-Regulated Protein 78 (Grp78) Confers Chemoresistance to Tumor Endothelial Cells under Acidic Stress

**DOI:** 10.1371/journal.pone.0101053

**Published:** 2014-06-25

**Authors:** Fernanda Visioli, Yugang Wang, Goleeta N. Alam, Yu Ning, Pantelis V. Rados, Jacques E. Nör, Peter J. Polverini

**Affiliations:** 1 Department of Biologic and Materials Sciences, University of Michigan School of Dentistry, Ann Arbor, Michigan, United States of America; 2 Department of Conservative Dentistry, Universidade Federal do Rio Grande do Sul School of Dentistry, Porto Alegre, Rio Grande do Sul, Brazil; 3 Department of Cariology, Restorative Sciences, and Endodontics, University of Michigan School of Dentistry, Ann Arbor, Michigan, United States of America; 4 Department of Periodontics and Oral Medicine, University of Michigan School of Dentistry, Ann Arbor, Michigan, United States of America; University of Pittsburgh, United States of America

## Abstract

**Objectives:**

This study was designed to investigate the activation of the unfolded protein response (UPR) in tumor associated endothelial cells (TECs) and its association with chemoresistance during acidic pH stress.

**Materials and Methods:**

Endothelial cells from human oral squamous cell carcinomas (OSCC) were excised by laser capture microdissection (LCM) followed by analysis of UPR markers (Grp78, ATF4 and CHOP) using quantitative PCR. Grp78 expression was also determined by immunostaining. Acidic stress was induced in primary human dermal microvascular endothelial cells (HDMECs) by treatment with conditioned medium (CM) from tumor cells grown under hypoxic conditions or by adjusting medium pH to 6.4 or 7.0 using lactic acid or hydrochloric acid (HCl). HDMEC resistance to the anti-angiogenic drug Sunitinib was assessed with SRB assay.

**Results:**

UPR markers, Grp78, ATF4 and CHOP were significantly upregulated in TECs from OSCC compared to HDMECs. HDMECs cultured in acidic CM (pH 6.0–6.4) showed increased expression of the UPR markers. However, severe acidosis led to marked cell death in HDMECs. Alternatively, HDMECs were able to adapt when exposed to chronic acidosis at pH 7.0 for 7 days, with concomittant increase in Grp78 expression. Chronic acidosis also confers drug resistance to HDMECs against Sunitinib. Knockdown of Grp78 using shRNA resensitizes HDMECs to drug treatment.

**Conclusions:**

UPR induction in ECs under acidic pH conditions is related to chemoresistance and may contribute to therapeutic failures in response to chemotherapy. Targeting Grp78, the key component of the UPR pathway, may provide a promising approach to overcome ECs resistance in cancer therapy.

## Introduction

Antiangiogenic therapy has emerged as a promising adjuvant approach to cancer treatment. However, evidence has shown that tumor associated endothelial cells (TECs) are more resistant to antiangiogenic therapies compared to endothelial cells from normal tissues (NECs) [1]. This resistant phenotype might be acquired by adaptation to stressful conditions in the tumor microenvironment (TME) [2].

TME is characterized by acidosis, low glucose, amino acid deficiency and hypoxia [3], [4]. A common response of cells to stress in the TME is the activation of the unfolded protein response (UPR), a protective mechanism that cells use to overcome endoplasmic reticulum (ER) stress. Different pathological insults can result in either acute or chronic ER stress [5]. Acute stress, if not resolved promptly, can lead to cell apoptosis. In contrast, chronic stress requires extended activation of the UPR, which enables cells to adapt and survive [6], [7].

The UPR is mediated through the activation of three ER transmembrane sensors.

PKR-like endoplasmic reticulum (ER) kinase (PERK), activating transcription factor 6 (ATF6) and inositol-requiring enzyme 1 (IRE1). These proteins are maintained in an inactive state through interaction with the chaperone, glucose-regulated protein 78 kDa (Grp78). Accumulation of unfolded proteins within the ER prompts dissociation of Grp78 from these sensors, and consequent activation of the UPR [8]. PERK activation leads to eukaryotic initiation factor 2α (eIF2α) phosphorylation, which inhibits general protein translation and reduces the load of nascent proteins being directed into the ER. PERK also induces ATF4, a transcription factor which upregulates ER chaperones (such as Grp78 and Grp94). Additionally, C/EBP homologous protein (CHOP) may be induced, which is known to promote apoptotic cell death [9], [10]. After being dissociated from Grp78, ATF6 translocates to the Golgi, where it is cleaved to the p50 active form. Active ATF6 translocates to the nucleus and regulates expression of genes, such as Grp78, protein disulphide isomerase (PDI) and X box-binding protein 1 (XBP1). The third ER stress sensor, IRE1, is responsible for the unconventional splicing of XBP1 mRNA. This alternatively spliced XBP1 encodes a transcription factor that targets diverse genes including chaperones [11]. Therefore, each one of the ER sensors activates a transcription factor, which induces proteins that aim to counteract ER stress and promote survival.

A common finding in a variety of malignant tumors is an increased expression of Grp78 [12], [13], [14]. Upregulation of Grp78 has been associated with chemoresistance to different drugs, such as adriamycin in squamous carcinoma cells [15]; temozolomide in glioma cells [16]; cisplatin in melanoma cells [17] and etoposide and temozolomide in TECs [18].

Although the relationship between UPR activation and chemoresistance in cancer cells is well-established, the effects of UPR on drug resistance of TECs need to be further investigated. The aim of this study was to assess the levels of UPR in ECs from the microvasculature of oral squamous cell carcinomas (OSCC) and to investigate how an acidic environment contributes to the chemoresistance of ECs. We report here that the UPR pathway is activated in ECs in response to acidic environment. Also, the key component of the pathway, Grp78 is, at least partially responsible for the drug resistance of ECs. Targeting Grp78 may provide a promising regimen towards sensitizing TECs to antiangiogenic drugs.

## Materials and Methods

### cell culture

Primary human dermal microvascular endothelial cells HDMECs (purchased from Lonza, Walkersville, MD) were cultured in endothelial growth medium EGM2-MV (Lonza, Walkersville, MD) at 37°C with 5% CO_2_. Medium pH was adjusted with 1 M lactic acid (Sigma-Aldrich, St. Louis, MO) or 1 M HCL (Sigma-Aldrich, St. Louis, MO). Tumor cell lines used in this study were received as gifts, human oral squamous cell carcinoma cell line UM-SCC-81B from Dr. Thomas E. Carey (Departments of Otorhinolaryngology and Pharmacology, University of Michigan Medical School, Ann Arbor, MI) and glioblastoma cell line U-87 from Dr. Yi Sun (Department of Radiation Oncology, University of Michigan, Ann Arbor, MI). Cell lines were mantained in Dulbecco’s modified Eagle’s medium (DMEM; Invitrogen, Carlsbad, CA) supplemented with 10% fetal bovine serum (FBS), 1% L-glutamine and 0.05% penicillin/streptomycin (Invitrogen) at 37°C with 5% CO_2_ [19], [20], [21], [22], [23], [24]. Conditioned medium (CM) was collected from tumor cells cultured in endothelial cell basal medium (EBM) for 48-hours under low oxygen condition. Collected CM was supplemented with glucose at 1.0****g/L (5.55 mM) to rule out glucose deprivation-induced UPR. The antiangiogenic drug Sunitinib was purchased from LC Laboratories (Woburn, MA).

### Lentivirus infection

Lentiviruses expressing short hairpin RNA (shRNA) constructs for Grp78 (Vector Core, University of Michigan) were generated in human embryonic kidney cells (293T) transfected by the calcium phosphate method. Scrambled oligonucleotide sequences (scshRNA) were used as controls. Virus containing supernatants were collected 48 hours post-transfection and used to infect HDMECs.

### Sulforhodamine B assay

Sulforhodamine B (SRB) cytotoxicity assays were done as previously described [25]. Briefly, cells were seeded at 2×10^3^ cells per well of 96-well plates, allowed to attach overnight, and treated with Sunitinib for 72 hours. Cells were fixed with 10% trichloroacetic acid, stained with 0.4% SRB (Sigma-Aldrich, St. Louis, MO) in 1% acetic acid, and plates were read in a microplate reader at 560 nm. Test results were normalized against drug-free controls. Data were obtained from triplicate wells per condition and is representative of at least three independent experiments.

### Western Blot

Cell lysates were resolved by SDS-PAGE and transferred to a PVDF membrane. Membranes were probed overnight at 4°C with the following antibodies from Santa Cruz Biotechnology (Santa Cruz, CA): rabbit anti-Grp78 (clone Sc-13968), rabbit anti-ATF4 (clone Sc-22800), rabbit anti-CHOP (clone Sc-575), mouse anti-β-actin (clone Sc-47778), αTubulin (Sc-8035); and from Cell Signaling (Danvers, MA): rabbit anti-*p*-eIF2α (clone 3597), rabbit anti-elF2α (clone 9722), mouse anti-caspase 7 (clone c7). Proteins were detected using peroxidase conjugated secondary antibodies and bands were visualized using enhanced chemiluminescence (Amersham, Sunnyvale, CA).

### Cell cycle analysis

HDMECs were trypsinized, washed with PBS twice and then fixed with 70% ethanol on ice for 1 h. The fixed cells were spun down and resuspended in PBS. After incubation with ribonuclease RNase A (25 µg/mL) at 37°C for 30 minutes, the cell suspension was stained with propidium iodide and followed by analysis on a FACSCalibur flow cytometer (BD Biosciences, San Jose, CA) in the Flow Cytometry Core, University of Michigan Comprehensive Cancer Center.

### Laser capture microdissection

Tissue sections of OSCCs and normal oral mucosa were mounted on glass foiled pen slides for laser capture microdissection (LCM) and stained with Hematoxylin. A two-step process was used for cell collection using a microdissection microscope (Leica AS LMD, Leica) with a pulsed 337-nm UV laser by a trained pathologist (FV). ECs were dissected after removal of blood cells from capillaries. Approximately 5000 ECs were retrieved from each sample. The RNA from independent tumors was analyzed by RT-PCR and real-time PCR for comparison to normal mucosa and HDMEC samples.

### Real-Time PCR

Total RNA of ECs from tissue sections obtained by LCM method was extracted using TRIzol reagent (Invitrogen, Carlsbad, CA) and purified with RNeasy Minikits (Qiagen, Valencia, CA) according to the manufacturers’ protocols. A cDNA library was prepared with WTA2, TransPlex Complete Whole Transcriptome Amplification Kit (Sigma-Aldrich, St. Louis, MO), which was then amplified using a universal end primer.

To harvest RNA from HDMECs, the RNeasy Minikit (Qiagen, Valencia, CA) was used and cDNA was made according to the manufacturer’s instructions with Verso cDNA kit (ThermoScientific, Waltham, MA).

Quantitative real-time (qRT-PCR) was performed with the following primers: Grp78 (Hs99999174_m1), ATF4 (Hs00909569_g1), CHOP (Hs01090850_m1) and β-tubulin (Hs 03929064_g1) using Gene Expression Assays reagents (Applied Biosystems, Carlsbad, CA). All reactions were done in triplicates and normalized to β-tubulin.

### RT- PCR

Reverse transcription PCR (RT-PCR) analysis of spliced and unspliced XBP1 was performed with a single human-specific primer pair: ACACGCTTGGGAATGGACAC (sense) and CCATGGGAAGATGTTCTGGG (antisense). Also, the following primer sequences were used: GAPDH, CATGGCCTCCAAGGAGTAAG (sense) and AGGGGTCTACAGGCAACTG (antisense); VEGFR2, AGCGATGGCCTCTTCTGTAA (sense) and ACACGACTCCATGTTGGTCA (antisense); E-cadherin, TGCCCAGAAAATGAAAAAGG (sense) and GGATGACACAGCGTGAGAGA (antisense). For XBP1 splicing analysis, amplicons were visualized with a Qiaxcel (Qiagen, Valencia, CA) automated nucleic acid fragment analyzer using a high resolution cartridge on the M500 setting using a 15 bp–1 kb alignment marker and a 50 bp–800 bp size marker.

### Immunofluorescence

Sections from the same tumors and samples from normal oral mucosa biopsies used for LCM were deparaffinized and rehydrated, and incubated in low pH antigen-retrieval solution (Dakocytomation; Dako, Carpinteria, CA) for 20 minutes at 95°C. Double staining was performed using antibodies against CD31 (Clone JC70A, Dako, Carpinteria, CA) and Grp78 (clone H-129, SantaCruz Biotechnology, Santa Cruz, CA). Alexa Fluor 488 or Alexa Fluor 647 (Molecular Probes, Eugene, OR) conjugated sencondary antibodies were used. Slides were counterstained with 3.5 µM DAPI (Sigma-Aldrich, St. Louis, MO), and mounted with Fluorescent Mounting Media (Dako, Carpinteria, CA). Images were taken with Olympus IX70 microscope (Tokyo, Japan) and analyzed by *ImageJ* software (National Institute of Health, Bethesda, MD, USA). Only CD31 positive cells lining tubular structures were considered blood vessels. Tissue samples were obtained from the University of Michigan School of Dentistry tissue core [19].

### Statistical analyses

For statistical comparison of experimental groups, *t*-test, one-way ANOVA and two-way ANOVA were used, followed by Bonferroni post-hoc test, using Software *Statistical Package for the Social Sciences* version 19.0 (SPSS Inc., Chicago, IL, EUA) and GraphPad Prism 5.0 (La Jolla, CA). Statistical significance was determined when p<0.05.

## Results

### UPR is activated in the vasculature of human tumors

**Figure 1:**
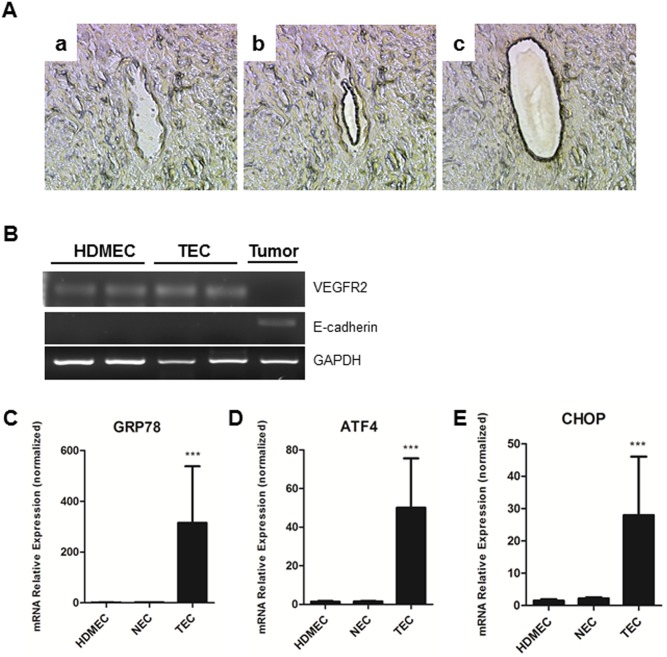
**In A**, LCM retrieval of endothelial cells from paraffin embedded tissue sections (a - before dissection, b - removal of blood cells, c- capture of endothelial cells). **B**, RNA purity control, using VEGFR2, a marker for endothelial cells, and E-cadherin, a marker for cells of ectodermal origin. **C, D, E**, real-time PCR to quantify Grp78, ATF4 and CHOP expression in TEC compared to NEC and primary endothelial cells. Specimens were obtained from human oral squamous cell carcinoma biopsies. Data presented from real-time PCR experiments reflect the expression levels of Grp78, ATF4 and CHOP normalized to β-tubulin. Values are means ± SD. Columns, means of individual experiments; Bars, SD. ***p<0.001.

To assess the UPR in tumor associated endothelial cells (TECs) *in vivo*, LCM was used to retrieve endothelial cells (EC) lining the blood vessels from human oral squamous cell carcinoma (OSCC) samples (Figure 1A). To confirm the purity of the TECs collected, RT-PCR was performed using VEGFR2 as a marker for ECs and E-cadherin as a marker for tumor cells. As it is shown in Figure 1B, VEGFR2 was detected in TECs, while there was no E-cadherin band suggesting that contamination from tumor cells is neglectable. Notably, Grp78 mRNA expression levels were ∼300-fold higher in TECs compared to endothelial cells from normal oral mucosa (NEC) and primary human dermal microvascular endothelial cells (HDMECs) (Figure 1C). To confirm if UPR is active in TECs, two other UPR makers (ATF4 and CHOP) were evaluated with qPCR and the results showed there was about 45-fold increase for ATF4 and 25-fold increase for CHOP (Figures 1D and E, respectively).

**Figure 2:**
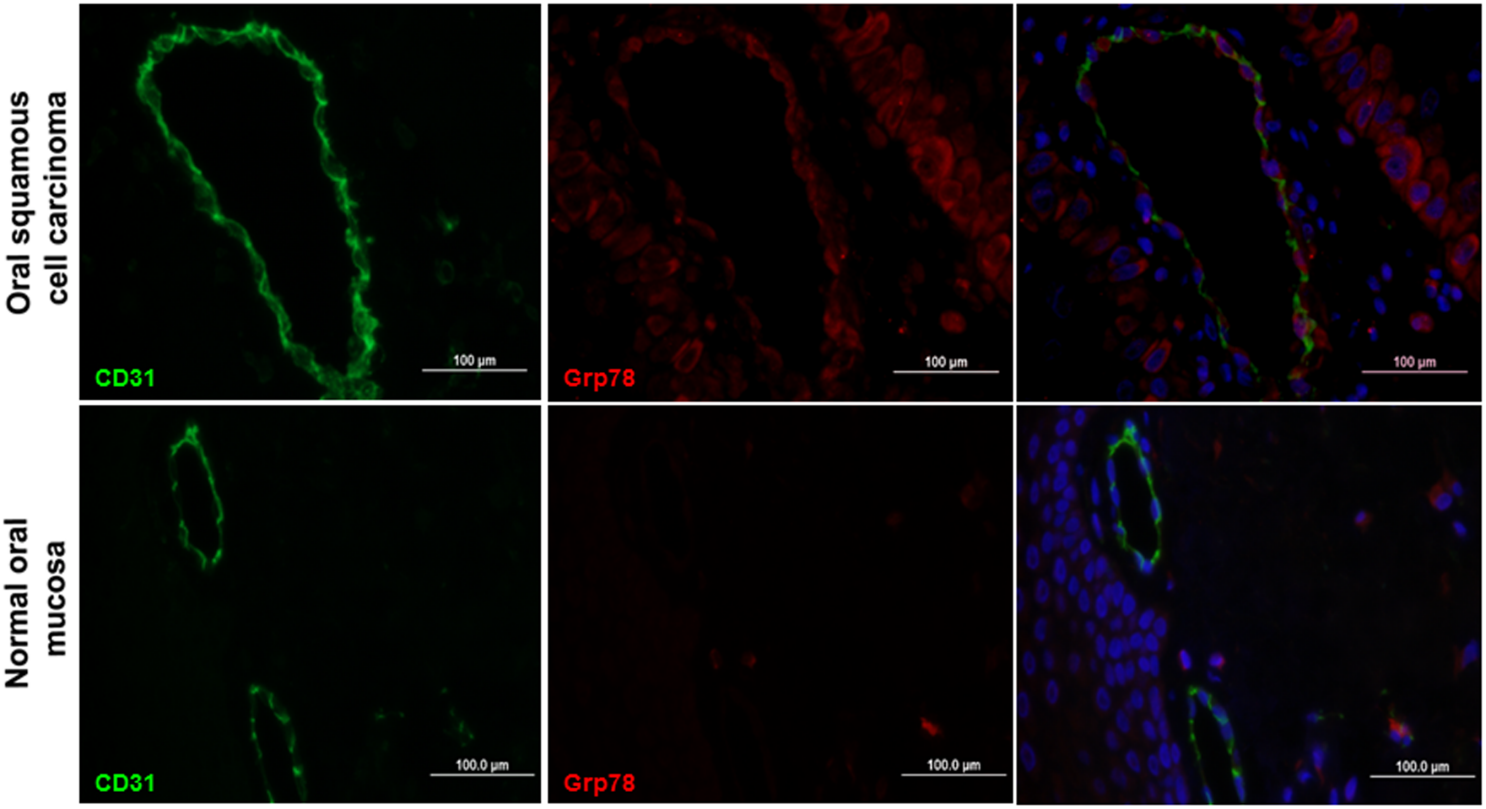
Grp78 expression in tumor vasculature of human oral squamous cell carcinomas. Histological sections of oral squamous cell carcinoma tissues (top) or normal oral mucosa tissues (bottom) were stained with anti-Grp78 antibody (red), anti-CD31 antibody (green), and 4,6-diamidino-2-phenylindole (DAPI; blue) nuclear staining; the images are merged in the last panel. Bar = 100 µm.

To address whether this observation is valid *in situ*, i.e. the vasculature of tumor tissues, sections of OSCC were immunostained for both Grp78 (red) and the endothelial cell marker CD31 (green), with DAPI (blue) staining the nuclei. Merged images confirmed that Grp78 was highly expressed in both the tumor vasculature and carcinoma cells. By contrast, normal oral mucosa tissues exhibited CD31-positive (green) blood vessels but minimal labeling for Grp78 (Figure 2). Merged images showed no Grp78 expression in vasculature of normal oral mucosa. These data confirm that Grp78 is preferably expressed in the tumor cells and TECs.

### Acidic tumor conditioned medium induces UPR in ECs

**Figure 3:**
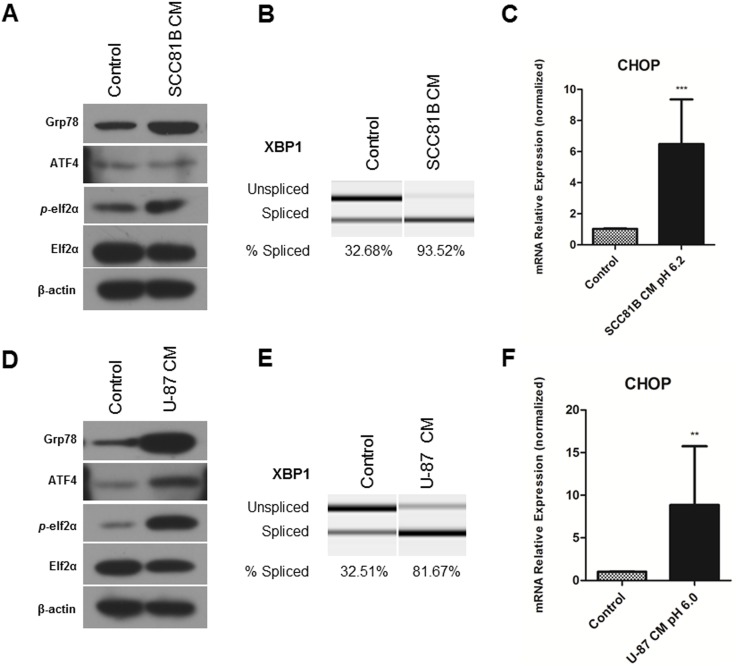
Tumor conditioned medium induces UPR in endothelial cells. Primary human endothelial cells, HDMECs, are maintained in conditioned medium obtained from oral squamous cell carcinoma UM-SCC-81B cell line (**A, B, C**) or U-87 glioblastoma cell line (**D, E, F**), HDMECs kept at regular medium served as control. Cell lysates were collected for western blot analysis, β-actin expression was used as a loading control. **A, D**, Treatment with CM increased protein expression of UPR markers: Grp78, ATF4, *p*-elf2α at 48 hours (western blot analysis). **B, E**, Exposure to CM increased XBP1 mRNA splicing levels at 48 hours. **C, F**, CM treatment upregulated CHOP mRNA levels. RNA was collected using Qiagen RNAeasy kit, reverse transcribed and analyzed using RT-PCR. Values are means ± SEM. Columns, means of individual experiments; Bars, SEM.**p<0.01, ***p<0.001.

To investigate if increased UPR levels in TECs are caused by the tumor microenvironment, we analyzed the effects of tumor conditioned medium (CM) on primary human dermal microvascular endothelial cells (HDMECs). The CM was prepared from the UM-SCC-81B oral squamous cell carcinoma cell line cultured under hypoxia to mimic the tumor microenvironment conditions. The CM was supplemented with glucose to 5.55 mM, the same concentration of regular endothelial medium to avoid glucose deprivation in ECs. After treatment with CM for 48 hours, HDMECs showed strong upregulation of UPR markers: Grp78, phosphorylation of elF2α and increased cleavage of XBP1 mRNA (Figures 3A and B) compared to ECs kept at regular medium for the same time. CHOP was also increased in ECs treated with CM from tumor cells as shown from qPCR in Figure 3C. These experiments were repeated with a second tumor model, the U-87 glioblastoma cell line, to exclude a cell-specific response (Figures 3D, E and F).

**Figure 4:**
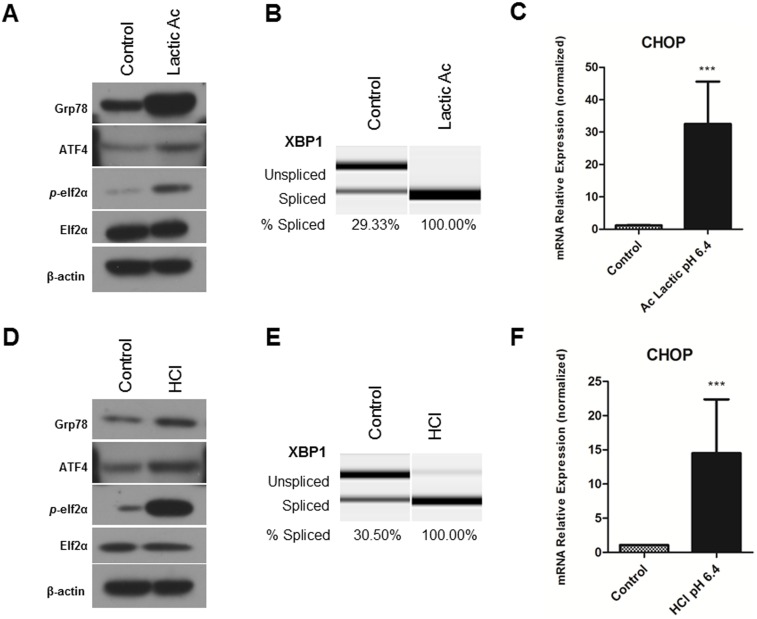
Acidic stress induces UPR in endothelial cells. Primary human endothelial cells, HDMECs, were maintained in acidic cultures for 48 hours; pH was adjusted by lactic acid (**A, B, C**) or HCl (**D, E, F**), HDMECs kept at regular pH 7.5 medium served as control. Cell lysates were collected for western blot analysis, β-actin expression was used as a loading control. **A, D**, Treatment with low pH increased protein expression of UPR markers: Grp78, ATF4, and *p*-elf2α at 48 hours (western blot analysis). **B, E**, Exposure to low pH increased XBP1 mRNA splicing levels at 48 hours. **C, F**. Acidic stress upregulated CHOP mRNA levels. RNA was collected using Qiagen RNAeasy kit, reverse transcribed and analyzed using RT-PCR. Values are means ± SEM. Columns, means of individual experiments; Bars, SEM.***p<0,001.

CM produced under hypoxia was found to be strongly acidic. The final pH of CM was 6.2 from UM-SCC-81B cells and 6.0 from U-87 cells. Considering that the extracellular milieu of solid malignant tumors is commonly acidic, we next analyzed specificity of endothelial cell response to acidic pH. HDMECs were treated in medium with a pH of 6.4 adjusted with lactic acid. After 48 hours in pH 6.4 medium, HDMECs showed strong upregulation of UPR markers proteins: Grp78, ATF4, phosphorylation of elF2α and increased cleavage of XBP1 mRNA (Figures 4A and B). Again, mRNA levels of CHOP were increased in acidic medium (Figure 4C). In addition, acidification with hydrochloric acid (HCl) was done to exclude cell-specific response and to confirm the results obtained from lactic acid treatments. As shown in Figure 4D to 4F, the response of ECs to acidic condition induced with HCl is similar to that induced with lactic acid.

### Mild acidic stress induces Grp78 expression and allows ECs to adapt and survive

**Figure 5:**
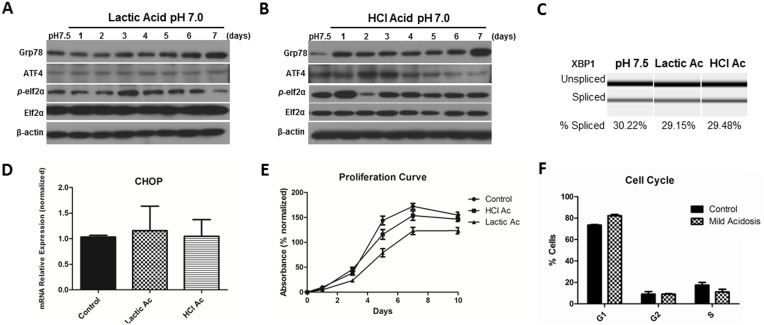
Mild acidic stress induces Grp78 expression mainly and allows endothelial cell adaptation. HDMECs were maintained in acidic cultures (pH 7.0) for seven days; pH was adjusted by lactic acid (**A**) or HCl (**B**), HDMECs kept at regular pH 7.5 served as control. Cell lysates were collected for western blot analysis, β-actin expression was used as a loading control; Grp78 expression increased over time. **C**, **D**, RNA was collected using Qiagen RNAeasy kits. XBP1 mRNA splicing and CHOP mRNA levels remained unaltered after exposure to mild acidic stress for seven days. Values are means ± SEM. Columns, means of individual experiments; Bars, SEM. **E**, Proliferation rates were determined by SRB assay. Values are means ± SD. Columns, means of individual experiments; Bars, SD. **F**, HDMECs exposed to pH 7.0 for seven days and HDMECs kept at pH 7.5 for same period were stained with propidium iodide and cell cycle was analyzed by flow cytometry. No difference in cell cycle distribution was observed. Values are means ± SD. Columns, means of individual experiments; Bars, SD.

We have demonstrated that acidic pH is a UPR inducer, however HDMECs were not able to survive under such acute stress and massive cell death occurred after 48 hours at pH 6.0–6.4 (data not shown). Therefore, the effects of mild acidic stress (which reflects conditions in the tumor microenvironment) were used during the following experiments. Mild acidic stress was induced by adjusting medium pH to 7.0 with lactic acid or HCl. During the 7-day treatment, Grp78 protein levels increased over time (Figures 5A and B). No significant changes were observed in XBP1 mRNA splicing levels (Figure 5C) or CHOP mRNA levels (Figure 5D). Under mild acidic stress HDMECs were able to proliferate slightly slower than cells in regular pH medium (Figure 5E), but no significant changes were observed in cell cycle distribution (Figure 5F). These results suggest that HDMECs may adapt to mild acidic stress by upregulation of Grp78 levels.

### Exposure to mild acidic stress results in a chemoresistance phenotype

**Figure 6:**
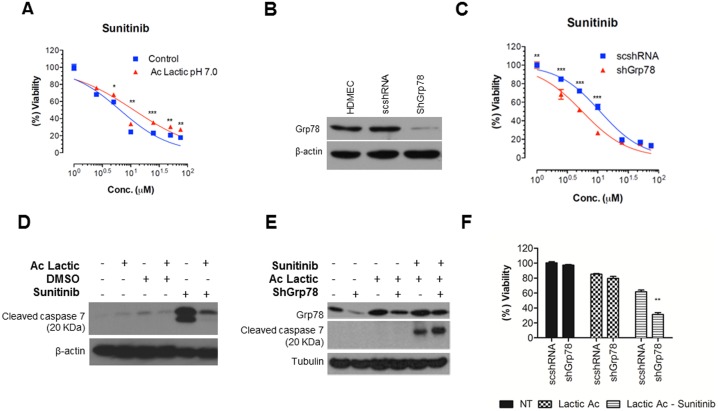
Chronic exposure to mild acidic stress results in a chemoresistance phenotype through Grp78 expression. **A**, HDMECs cultured under acidic stress for 7 days, with medium pH adjusted to 7.0 with lactic acid and HDMECs maintained at regular pH 7.5 for seven days (controls) were exposed to Sunitinib at different doses or the vehicle control (DMSO) for further 72 h and then examined for cell viability using the SRB assay. Vehicle treatment represents 100% viability. **B**, HDMECs were infected with control ShRNA (scshRNA) or ShRNA specifically targeted against human Grp78 (ShGrp78) or left uninfected; Four days after infection, cells were harvested for western blot analysis and probed for Grp78 and β-actin. **C**, Seven days after mild acidic stress (pH 7.0), scshRNA and ShGrp78 cells were treated with either vehicle or Sunitinib for further 72 h and then examined for cell viability using the SRB assay. Vehicle treatment represents 100% viability. **D**, HDMECs were maintained in acidic cultures (pH 7.0) for 7 days, HDMECs at regular pH 7.5 served as control. At day 7, cells were treated with either DMSO (Vehicle, 1%), or Sunitinib (0.25 µM) for an additional 48 hours after which cell lysates were collected for western blot analysis and probed for caspase 7 and β-actin. Previous exposure to acidic stress resulted in lower levels of cleaved caspase 7. **E**, ScshRNA and ShGrp78 cells were treated with Sunitinib (0.25 µM) for 48 hours after which cell lysates were collected for western blot analysis and probed for caspase 7 and α-tubulin. We observed increased caspase 7 activation in ShGrp78 cells treated with Sunitinib. **F**, Survival analysis showed increased cell death in ShGrp78 cells treated with Sunitinib. Values are means ± SEM. Columns, means of individual experiments; Bars, SEM. Significance is calculated by comparing drug-treated cells and vehicle control-treated cells. *p<0.05, **p<0.01, ***p<0.001.

It has been shown that Grp78 plays a role in chemoresistance of tumor cells [15]–[17], so we hypothesized that Grp78 upregulation under mild acidic stress may contribute to chemoresistance of ECs. To test this hypothesis, the sensitivity of HDMECs to an antiangiogenic drug was studied under acidic stress. After exposing HDMECs to pH 7.0 for up to 7 days, cells were treated with the antangiogenic drug Sunitinib (1–75 µmol/L) and DMSO (vehicle; 0.1%) for 72 hours. Cell survival was determined using SRB assay. The results show that pretreatment of HDMECs with chronic acidic stress is able to increase EC50 from 6.48 µM to 11.31 µM for Sunitinib treatment, suggesting that chronic stress confers protective effects (Figure 6A).

To determine whether Grp78 overexpression contributes to chemoresistance, HDMECs were infected with lentivirus expressing shRNA against human Grp78 (shGrp78) with scrambled shRNA as control (scshRNA). Four days after infection, cell samples were analyzed for Grp78 expression by western blot. As shown in Figure 6B, the expression of Grp78 was significantly reduced, and as expected scshRNA showed no effect (Figure 6B). To determine the role of Grp78 in regulating chemoresistance of HDMECs, both shGrp78 and scshRNA cells were kept in acidic medium for 7 days and then treated with Sunitinib (1–75 µmol/L) for addtional 72 hours. We observed that Grp78 knockdown makes HDMECs more sensitive to drug treatment, EC50 of Sunitinib was decreased from 11 µM to 5.5 uM (Figure 6C). In conclusion, knocking down Grp78 resensitizes HDMECs to Sunitinib treatment. These results corroborate the notion that chronic stress confers drug resistance to HDMECs by increasing Grp78 expression.

### Acidic stress suppresses drug-mediated cleavage of caspase 7

It has been shown that caspase 7 is associated with genotoxic drug-induced cell death and Grp78 can directly inhibit its activation [26], [27]. Upon induction of apoptosis, procaspase 7 is processed into an active 20-kDa subunit. To study the mechanisms involved in Grp78-induced chemoresistance, we analyzed the impact of preconditioning with mild acidic stress on caspase 7 cleavage. We found that cleavage of caspase 7 was significantly inhibited in HDMECs preexposed to chronic mild acidic stress prior to drug treatment, suggesting that chronic stress can protect HDMECs from apoptosis by inhibiting the production of active caspase 7 (Figure 6D).

Futhermore, shGrp78 cells showed increased caspase 7 activation along with decreased survival after Sunitinib treatment as compared to scshRNA cells (figure 6E and F). These results strengthen the central role of GRP78 in promoting chemoresistance through reducing caspase 7 cleavage.

## Discussion

The tumor microenvironment (TME) is characterized by extracellular acidosis, glucose deprivation (GD) and low oxygen tension [3], [4]. We have previously demonstrated that GD promotes angiogenesis by inducing expression of angiogenic mediators, such as VEGF, FGF and IL6 through the activation of UPR [19]. Here, expression of UPR markers in ECs from human OSCC was evaluated using LCM to analyze endothelium gene expression *in situ*. The data obtained shows that Grp78 mRNA expression levels were ∼300-fold higher in ECs retrieved from human tumors, compared to normal human ECs. Immunofluorescence staining with CD31 and Grp78 confirmed the overexpression of Grp78 in TECs and carcinoma cells compared to the vasculature and epithelium from normal oral mucosa.

Recently it has been reported that TECs are more chemoresistant to drugs when compared to NECs [1]. Virrey and his colleagues reported increased expression of Grp78 in human ECs derived from blood vessels of malignant glioblastoma tumors, and Grp78 expression showed positive correlation with chemoresistance to CPT-11, etoposide, and temozolomide [18]. Since the emergence of angiogenesis as a potential target for tumor therapy, several antiangiogenic therapies have been developed. However, antiangiogenic therapies have not produced the sustained antitumor benefit, once envisioned. This is in part, due to the fact that the biology of TECs is more complex than previously thought [2], and that many early antiangiogenesis studies had not taken into account the influence of the TME on the angiogenic phenotype. Acidic pH, a feature of the TME, can potentially modify the endothelial phenotype. We found that the increased expression of Grp78 in ECs after exposure to chronic acidic stress is related to a drug-resistant phenotype. The acidic pH of the TME is well known to increase chemoresistance in tumor cells [28], [29], [30]. To our best knowledge this is the first report of acidic stress contributing to EC chemoresistance through Grp78 induction. Exposing ECs to a pH of 6.4 induces acute UPR and ultimately results in cell death at 48 hours of treatment, while mild acidic stress triggers an adaptive UPR with progressive increase in Grp78 expression.

In our study, different stimuli produced distinct UPR responses; UM-SCC-81B CM was not able to increase ATF4 expression, unlike U-87 CM treatment. During chronic stress induction, lactic acid adjusted medium did not increase ATF4 levels, in comparison to HCl treatment. In addition, increased expression of Grp78 after HCl treatment occurred after 24 hours, whereas medium adjusted with lactic acid took longer to induce significant Grp78 expression. While the reasons for these disparaties are unknown, a striking common response to both stimuli is increased Grp78 levels.

Depending on the intensity and duration of stress, the UPR response is structured to enable cells to either adapt to stress or to undergo apoptosis. Considering that the UPR is linked to several pathological conditions, it is important to understand how the molecular decisions, which allow cells to adapt to stress rather than undergo apoptosis, are made. This knowledge is essential in developing therapies that on the one hand allow cells to avoid apoptosis in diseases such as neurodegenerative disorders and diabetes, while on the other hand, program cells to undergo apoptosis for diseases such as cancer. Rutkowski et al. [7] demonstrated that a possible mechanism of adaptation to mild stress is the consistent expression of proteins that facilitate survival, in particular ER chaperones, without persistence of proapoptotic proteins such as CHOP, possibly due to selective intrinsic instabilities of mRNAs and proteins. Our results are in agreement with Rutkowski et al. [7], because HDMECs seem to adapt to chronic stress induced by acidic pH by upregulating Grp78, an ER chaperone with pro-survival functions.

Chemoresistance was abrogated by Grp78 knockdown, demonstrating that Grp78 plays a key role in the resistant phenotype of ECs under acidic stress. The exact mechanism by which Grp78 protects cells from death is not known. Whereas the majority of Grp78 resides in the ER lumen, it has been shown that a fraction of Grp78 exists as an ER transmembrane protein, which could explain how Grp78 can directly inhibit the activation of caspase 7, an executor caspase activated by both ER stress and genotoxic drugs [26], [27]. Ours results support the notion that pretreatment with acidic stress decreases the cleavage of caspase 7 in HDMECs after drug treatment and protects HDMECs from apoptosis.

The effects of acidic stress on ER homeostasis are poorly understood. There are several possible ways by which acidic pH may induce ER stress, including direct inactivation of enzymes involved in ER protein processing, denaturation of proteins, and inhibition of the ER Ca2þ/ATP-ase [31], [32]. Previous reports have shown the effects of acidic pH in coronary ECs, in the context of myocardial ischemia. The main pathway of acidity-induced apoptosis consists of Ca^2+^ leak from the ER [33]. Therefore, acidic stress may cause ER stress by decreasing ER Ca^2+^ levels, which is required for optimum protein folding since many of chaperones are Ca^2+^-dependent [13]. Additionally, Kumar et al. reported that acidic pre-conditioning (APC) suppresses apoptosis in coronary ECs [34]. The concept of stress promoting resistance to cell death is in accordance with the preconditioning paradigm: almost any stress factor that is potentially harmful for cells can elicit a preconditioned state, i.e. increased resistance to the stress, when applied in small quantities [35]. The mild acidic stress conferring chemoresistance to HDMECs further supports this notion.

This work illustrates the complexities of effects of the TME on tumor angiogenesis, highlighting acidic stress as an important feature of the TME that modifies the EC phenotype. Also, this work suggests that UPR-mediated endothelial resistance may results in therapeutic failures. The results indicate that UPR response promoted by the TME is in favor of tumor growth and resistance to chemotherapy. Targeting UPR components, such as Grp78 may provide a promising alternative in cancer treatment.
